# Relationship of Income and Health Care Coverage to Receipt of Recommended Clinical Preventive Services by Adults — United States, 2011–2012

**Published:** 2014-08-08

**Authors:** Jared B. Fox, Frederic E. Shaw

**Affiliations:** 1 Office of Health System Collaboration, Office of the Associate Director for Policy, CDC; 2 Center for Surveillance, Epidemiology, and Laboratory Services, CDC

Each year in the United States, an estimated 100,000 deaths could be prevented if persons received recommended clinical preventive care (1). The Affordable Care Act has reduced cost as a barrier to care by expanding access to insurance and requiring many health plans to cover certain recommended preventive services without copayments or deductibles. To establish a baseline for the receipt of these services and to begin monitoring the effects of the law, CDC analyzed responses from persons aged ≥18 years in the National Health Interview Survey (NHIS) for the years 2011 and 2012 combined. This report summarizes the findings for six services covered by the Affordable Care Act. Among the six services examined, three were received by less than half of the persons for whom they were recommended (testing for human immunodeficiency virus [HIV] and vaccination for influenza and zoster [shingles]). Having health insurance or a higher income was associated with higher rates of receiving these preventive services, affirming findings of previous studies (2). Securing health insurance coverage might be an important way to increase receipt of clinical preventive services, but insurance coverage is not all that is needed to ensure that everyone is offered and uses clinical services proven to prevent disease. Greater awareness of Affordable Care Act provisions among public health professionals, partners, health care providers, and patients might help increase the receipt of recommended services (3).

The analysis focused on responses to questions about the receipt of six clinical preventive services recommended by the U.S. Preventive Services Task Force (USPSTF) or the Advisory Committee for Immunization Practices (ACIP). The six preventive services are among dozens of services for adults covered without copayments or deductibles under certain health plans according to the Affordable Care Act,* and were selected for this analysis because the recommendations closely fit NHIS survey questions. The six were as follows: HIV testing, smoking cessation discussion, influenza vaccination, pneumococcal vaccination, tetanus vaccination, and zoster (shingles) vaccination. However, the recommendations and NHIS questions are not a perfect match. For example, cessation intervention is recommended for all forms of tobacco use, but respondents were only asked about receiving smoking cessation interventions. The fit between the NHIS questions and the recommendations varied among the six preventive services (Table 1).

NHIS is administered by in-person interviews to a nationally representative sample of the noninstitutionalized, U.S. civilian population. For this analysis, NHIS data from the sample adult core questionnaire in 2011 and 2012 were combined to increase sample sizes and improve reliability of estimates. In each household identified, one adult (aged ≥18 years) from each family was randomly selected to complete the questionnaire.† NHIS 2011 and 2012 adult core samples included 33,014 and 34,525 respondents, respectively, and the overall response rates were 66.3% and 61.2%.

Participants were asked whether they had health insurance at the time of the interview. They were considered uninsured if they reported currently not having private health insurance, Medicare, Medicaid, Children’s Health Insurance Program, a state-sponsored or other government-sponsored health plan, or a military plan. Respondents also were defined as uninsured if they had only a private plan that paid for one type of service (e.g., injury or dental care) or had only Indian Health Service coverage.§ Multiple imputations were performed on family income to account for missing responses to income questions.¶ NHIS data were adjusted for nonresponse and weighted to provide national estimates of insurance status and receipt of preventive care; 95% confidence intervals were calculated, taking into account the survey’s multistage probability sample design. Generalized linear modeling and the t-test were used to calculate prevalence ratios and statistical significances of differences in preventive services receipt between 1) persons who were insured and those who were uninsured, 2) those with current family incomes >200% of the federal poverty level (FPL) ($46,100 for a family of four in 2012**) and those with incomes ≤200% of the FPL, and 3) those with any private health insurance and those with only public coverage.

For the six services examined, prevalence of receipt of service was as follows: zoster vaccination, 17.9%; influenza vaccination, 39.4%; HIV testing, 41.7%; smoking cessation discussion, 52.0%; pneumococcal vaccination, 61.4%; and tetanus vaccination, 62.0% (Table 2). A higher percentage of adults with health insurance received five of six recommended clinical preventive services (all but HIV testing) compared with those who were uninsured (Table 2). Among those five services, the service receipt prevalence ratio for those with insurance compared with those without insurance ranged from 1.2 for tetanus vaccination to 3.4 for pneumococcal vaccination (Table 2). However, service receipt for persons with health insurance was <50% for three of six recommended clinical preventive services.

Persons with family incomes >200% of the FPL received five of six recommended clinical preventive services at a statistically significant higher prevalence compared with those with incomes below that threshold (Table 3). Among those five services, the service receipt prevalence ratio for those with family incomes >200% of the FPL compared with those with incomes ≤200% of the FPL ranged from 1.1 for pneumococcal vaccination to 1.9 for zoster vaccination (Table 3).

Persons with private health insurance received three of six recommended clinical preventive services at a higher prevalence, and three of six at a lower prevalence, compared with those with only public insurance (Table 4).

## Discussion

The findings in this report indicate that during 2011–2012, large portions of the adult population were not receiving recommended preventive care, those with insurance were more likely to receive recommended preventive services than those without coverage, and those with higher income were more likely to receive recommended care. This supports previously published studies, including one that found prevalence ratios in the range of 1–3 for those with insurance receiving recommended preventive services compared with those without coverage (2). However, even among persons with insurance and higher income, in this analysis, receipt of recommended preventive services was suboptimal.

This report could serve as a baseline for tracking the effects of the Affordable Care Act on the receipt of six preventive services. Although the law began to require certain plans to cover clinical preventive services in September 2010, the data from 2011–2012 provide a feasible baseline for measuring the law’s effects because 1) a high number of persons remained uninsured during 2011–2012, 2) there was little awareness of the preventive care provisions of the new law, and 3) many plans in existence before enactment of the Affordable Care Act were not subject to the preventive services provisions (4–6).

The findings in this report are subject to at least four limitations. First, this was a cross-sectional study, and associations between receipt of a service and other factors do not imply a causal relationship. Second, insurance coverage and income level are just two of many factors that might be associated with service receipt rates. This analysis does not include possible confounders such as education, health status, or other factors. Third, receipt of preventive services was self-reported and might be subject to recall bias. Finally, inferences from these results are limited by differences in time between when the questions were asked and when the services were received. For example, NHIS identifies whether the respondent is insured at the time of interview; however, depending on the service, NHIS asks whether the respondent received preventive care in the last 12 months, last 10 years, or ever during their lifetime. Currently uninsured respondents might have received preventive care during a time when they had insurance, or vice versa. In addition, NHIS is limited to noninstitutionalized civilians, excluding certain populations (e.g., the institutionalized and the military) that might be especially likely to receive recommended preventive services.

What is already known on this topic?

Rates of receipt of some clinical preventive services by adults are low, but higher for persons with insurance coverage or higher incomes. The Affordable Care Act’s expansions of health insurance access and coverage requirements for clinical preventive services were developed to increase access to health services to improve the health of the population.

What is added by this report?

Analysis of combined adult responses to the National Health Interview Survey in 2011 and 2012 indicated that persons with health insurance were more likely to have received five of six recommended preventive services than persons without insurance. However, regardless of insurance status, receipt was below 50% for three services and ranged from 17.9% for zoster vaccination to 62.0% for tetanus vaccination.

What are the implications for public health practice?

Increased insurance coverage might lead to a substantial increase in receipt of preventive care and improvements in population health. However, low rates of service receipt even among those with insurance suggest that additional efforts beyond insurance coverage expansion might be needed to increase offering and use of services.

All new private health plans, alternative benefit plans for the newly Medicaid eligible, and Medicare now provide coverage without copayments or deductibles for recommended clinical preventive services. By expanding access to insurance and requiring many plans to cover recommended clinical preventive services, the Affordable Care Act is expected to reduce barriers to receipt of recommended preventive care. The number of uninsured persons aged <65 years is expected to drop from 55 million in 2013 to 30 million in 2017 (7).

Lack of insurance, however, is not the only barrier to receiving services; a number of other factors likely will continue to inhibit receipt of preventive care. First, many persons are currently insured under “grandfathered” health plans not required to provide coverage without copayments or deductibles for all recommended preventive services (8). Second, other barriers, such as transportation costs and lack of a regular physician, might inhibit receipt of recommended preventive care. Finally, even after the Affordable Care Act is implemented fully, millions of persons are expected to remain uninsured (7). To date, about half of the 50 states have not yet implemented the law’s expansion of Medicaid, leaving an estimated 40% of their adult residents who have been uninsured in the last 2 years without access to affordable care (9). Studies have indicated that 60%–74% of children who are eligible for Medicaid are uninsured, in part as a result of failure to renew enrollment in Medicaid (10). Efforts to increase enrollment and coverage retention could help these populations maintain continuous coverage, thereby increasing receipt of preventive services and reducing avoidable complications from illness, long-term health care costs, and premature deaths (10).

## Figures and Tables

**TABLE 1:** Comparison of recommendations from the United States Preventive Services Task Force (USPSTF) and the Advisory Committee on Immunization Practices (ACIP) with questions regarding six recommended clinical preventive services in the National Health Interview Survey (NHIS) — United States, 2011–2012

Clinical preventive service (age group)	Recommendation	Question to NHIS participants	Key distinctions for this analysis of use of recommended services
HIV test (age 18–65 years)	HIV infection screening is recommended for persons aged 15–65 years. Screening is recommended for other age groups at increased risk. Recommended screening interval for the general population is not specified.*	To adults aged ≥18 years: “Except for tests you may have had as part of blood donations, have you ever been tested for HIV?”†	NHIS asks this question to those aged ≥18 years. Those aged 15–17 years are not included in the analysis.
Smoking cessation discussion (age ≥18 years)	Tobacco cessation interventions are recommended for those who use tobacco products. A recommended screening interval for the general population is not specified.*	To adults aged ≥18 years who currently smoke cigarettes every day or some days: “During the past 12 months, has a doctor or other health professional talked to you about your smoking?”	Adults who use tobacco only in forms other than cigarettes are not included in the analysis.
Influenza vaccination (age ≥18 years)	Annual vaccination against influenza is recommended for all persons aged ≥6 months.§	To adults aged ≥18 years: “During the past 12 months, have you had a flu shot?” and “During the past 12 months, have you had a flu vaccine sprayed in your nose by a doctor or other health professional?” A “yes” response to either question is coded as vaccination received.	This analysis focuses on adults aged ≥18 years.
Pneumococcal vaccination (age ≥65 years)	Pneumococcal vaccination is recommended for all persons aged ≥65 years and for persons with certain other risk factors aged <65 years.§	“Have you ever had a pneumonia shot?”†	This analysis focuses on those aged ≥65 years.
Tetanus vaccination (age ≥19 years)	Vaccination with Td booster (or 1-time dose of Tdap) for all adults aged ≥19 years.§	To adults aged ≥18 years: “Have you received a tetanus shot in the past 10 years?”	This analysis focuses on those aged ≥19 years for consistency with the recommendation for adults.
Zoster (shingles) vaccination (age ≥60 years)	Zoster vaccination is recommended for adults aged ≥60 years.§	To adults aged ≥50 years: “Have you ever had the zoster or shingles vaccine, also called Zostavax?”†	This analysis focuses on those aged ≥60 years for consistency with the recommendation for adults.

**Abbreviations:** HIV = human immunodeficiency virus; Td = tetanus and diphtheria; Tdap = tetanus toxoid, reduced diphtheria toxoid, and acellular pertussis.

* **Source:** USPSTF.

† At any age.

§ **Source:** ACIP.

**TABLE 2:** Percentage of adults in the recommended populations who received six clinical preventive services, by health insurance status — National Health Interview Survey, United States, 2011–2012

Clinical preventive service (age group)	Insured receiving service			Uninsured receiving service			Prevalence ratio, insured/uninsured*	(95% CI)	Total receiving service	
		
	No.	%	(95% CI)	No.	%	(95% CI)			%	(95% CI)
HIV test (ever) (age 18–65 years)	40,823	41.5	(40.7–42.2)	11,641	43.1	(41.9–44.3)	1.0†	(0.9–1.0)	**41.7**	**(41.1–42.4)**
Smoking cessation discussion (within 12 mos) (age ≥18 years)	8,935	59.1	(58.0–60.3)	3,497	32.7	(31.1–34.4)	1.8§	(1.7–1.9)	**52.0**	**(51.0–53.0)**
Influenza vaccination (within 12 mos) (age ≥18 years)	54,217	44.2	(43.6–44.7)	11,888	14.7	(13.9–15.4)	3.0§	(2.9–3.2)	**39.4**	**(38.9–40.0)**
Pneumococcal vaccination (ever) (age ≥65 years)	13,585	61.7	(60.6–62.7)	113	18.1	(9.1–27.0)	3.4§	(2.1–5.6)	**61.4**	**(60.3–62.4)**
Tetanus vaccination (within 10 years) (age ≥19 years)	51,872	63.7	(63.0–64.3)	11,431	53.7	(52.6–54.8)	1.2§	(1.2–1.2)	**62.0**	**(61.5–62.6)**
Zoster vaccination (ever) (age ≥60 years)	18,297	18.4	(17.6–19.2)	868	6.3	(4.2–8.4)	2.9§	(2.1–4.1)	**17.9**	**(17.1–18.7)**

**Abbreviations:** CI = confidence interval; HIV = human immunodeficiency virus.

* Generalized linear modeling was used to identify statistical significance of differences between insured and uninsured persons receiving service.

† p<0.015.

§ p<0.001.

**TABLE 3:** Percentage of adults in the recommended populations who received six clinical preventive services, by family income level — National Health Interview Survey, United States, 2011–2012

Clinical preventive service (age group)	Income >200% FPL receiving service			Income ≤200% FPL receiving service			Prevalence ratio, higher income/lower income*	(95% CI)
	
	No.	%	(95% CI)	No.	%	(95% CI)		
HIV test (ever) (age 18–65 years)	31,948	40.2	(39.4–40.9)	25,815	44.6	(43.5–45.7)	0.9†	(0.9–0.9)
Smoking cessation discussion (within 12 mos) (age ≥18 years)	6,068	53.5	(52.2–54.8)	6,404	50.4	(48.9–51.9)	1.1§	(1.0–1.1)
Influenza vaccination (within 12 mos) (age ≥18 years)	40,110	42.8	(42.2–43.4)	26,201	33.4	(32.6–34.3)	1.3†	(1.3–1.3)
Pneumococcal vaccination (ever) (age ≥65 years)	8,268	64.4	(63.1–65.6)	5,449	56.2	(54.5–57.9)	1.1†	(1.1–1.2)
Tetanus vaccination (within 10 years) (age ≥19 years)	38,893	65.0	(64.4–65.7)	24,840	56.6	(55.7–57.5)	1.1†	(1.1–1.2)
Zoster vaccination (ever) (age ≥60 years)	12,025	21.4	(20.4–22.4)	7,177	11.3	(10.3–12.3)	1.9†	(1.7–2.1)

**Abbreviations:** CI = confidence interval; HIV = human immunodeficiency virus; FPL = federal poverty level.

* Generalized linear modeling was used to identify statistical significance of differences betweeen persons at higher income level and lower income level receiving service.

† p<0.001.

§ p<0.005.

**TABLE 4:** Percentage of adults in the recommended populations who received six clinical preventive services, by source of health insurance coverage — National Health Interview Survey, United States, 2011–2012

Clinical preventive service (age group)	Private insurance receiving service			Only public insurance receiving service			Prevalence ratio, private/public*	(95% CI)
	
	No.	%	(95% CI)	No.	%	(95% CI)		
HIV test (ever) (age 18–65 years)	31,605	38.6	(37.8–39.3)	9,218	53.0	(51.6–54.3)	0.7†	(0.7–0.8)
Smoking cessation discussion (within 12 mos) (age ≥18 years)	5,399	55.3	(53.9–56.8)	3,535	65.8	(64.0–67.5)	0.8§	(0.8–0.9)
Influenza vaccination (within 12 mos) (age ≥18 years)	38,470	42.4	(41.8–43.1)	15,738	48.9	(47.9–49.9)	0.9§	(0.8–0.9)
Pneumococcal vaccination (ever) (age ≥65 years)	6,807	66.1	(64.8–67.4)	6,769	56.9	(55.3–58.4)	1.2§	(1.1–1.2)
Tetanus vaccination (within 10 years) (age ≥19 years)	36,917	65.7	(65.1–66.4)	14,946	57.9	(56.9–58.9)	1.1§	(1.1–1.2)
Zoster vaccination (ever) (age ≥60 years)	10,305	20.4	(19.4–21.4)	7,984	15.7	(14.6–16.7)	1.3§	(1.2–1.4)

**Abbreviations:** CI = confidence interval; HIV = human immunodeficiency virus.

* Generalized linear modeling was used to identify statistical significance of differences between persons with private insurance and only public insurance.

† p<0.05.

§ p<0.001.

## Footnotes

* For adults, the Affordable Care Act recognizes USPSTF (http://www.uspreventiveservicestaskforce.org) and ACIP (http://www.cdc.gov/vaccines/acip) as organizations whose clinical preventive service recommendations receive coverage without copayments and deductibles for certain health plans.

† Additional information available at http://www.cdc.gov/nchs/nhis/quest_data_related_1997_forward.htm.

§ Consistent with other population surveys conducted by U.S. federal agencies, CDC does not regard Indian Health Service coverage as health insurance for the purpose of identifying uninsured populations.

¶ Additional information available at http://www.cdc.gov/nchs/nhis/2011imputedincome.htm and http://www.cdc.gov/nchs/nhis/2012imputedincome.htm.

** Additional information available at http://aspe.hhs.gov/poverty/12poverty.shtml and http://www.census.gov/hhes/www/poverty/about/overview/measure.html.
